# President’s Address

**Published:** 1889-04

**Authors:** H. L. Moore


### COMMUNICATIONS.

### President's Address.

BY H. L. MOORE, D.D.S.

Read before the Mississppi Valley Dental Society, Cincinnati, March 6th, 1889.

It seems almost an empty formality for me to utter words of welcome on this occasion. The welcome that has always been extended to you by the former presidents of this, the oldest dental association in the world, has borne with it a genuine heartiness and earnestness which words of mine can but feebly echo.

But speaking as the representative of the grand old dental society with which we are united by the bonds of a common fraternity, I can with peculiar emphasis and significance extend to you the right hand of fellowship, and say that we are happy to greet you on this occasion.

Almost a half century has rolled around since the first meeting of this society. While the number in attendance does not increase to any great extent year after year, yet we have on the books of this society the names of dentists scattered throughout the world. Men whose articles on various subjects pertaining to our profession, are to be seen in medical and dental journals of the day, also whose text-books are used in the various universities and dental schools of the land.

Yet, friends, when we look around this company we do not see some of the old familiar faces that we have been in the habit of seeing year after year, whose attendance has brought new life and happiness as well as imparted knowledge and instruction, particularly to those of us who represent the younger membership of this society, the instruction and knowledge imparted by these grand men have been the means of straightening many a crooked path and smoothing many a rough place. I speak more particularly of Dr. Taylor and Dr. Keely—both taken away while yet in active duty. I cherish in my memory the happy thought that they are now with our heavenly Father, and that the good they have done will live after them. There is no end to the good they have done. It is not necessary to go into the history of either one of them. As you all well know Dr. Taylor was one of the founders of the second oldest dental school in the world, which has been in operation forty-three years and is now with the largest class in its history. My first recollection of Dr. Keely was while a student. As you all know he was authority on irregularities of the jaw, and teeth, and as has been his custom year after year, attended the annual meetings of this society. Dr. Keely imparted his knowledge and instruction so that we all have been greatly benefited by it. Both men were loved by the whole profession, they were happy in their family and professional relations and in doing good for others.

It is a law of our intellectual and moral being that we form our own real happiness in the exact proportion in which we contribute to the comfort and happiness of others. That only is the true philosophy which recognizes and works on the principle in daily life that—

> "Life Was Lent for Noble Deeds.
> There is a power to make each hour
> As sweet as Heaven designed it;
> Nor need we roam to bring it home,
> Though few there be to find it.
> We seek too high for things close by,
> And lose what Nature found us;
> For life hath here no charms so dear,
> As home and friends around us."

It has been said an author is known by his writings, a mother by her daughter, a fool by his friends, and all men by their companions. The force of example is powerful. Our tempers and habits are formed very much upon the model of those with whom we associate. From youth to old age man is a creature of circumstances, and his course of life is shaped very largely by his environments. But not only do external associations affect and determine character, but also the motives and purposes of life which we adopt and accept lend color and direction to our actions and indicate the paths which our feet shall tread.

Thus it happens that a man's chosen associations are at once an index and a prophecy of what he is, and a faithful foretelling of what he will be.

Coming together as we do year after year we are united for the high and lofty purposes which are the objects of our organizations; we come together not as strangers, dubious of one another, entering upon some untried and experimental undertaking, but as professional brothers, moved by a common, noble, and well-understood purpose.

We embrace in the purpose of our organization all those beautiful sentimentalities which cluster and cling around the idea of friendship and fraternity, and, at the same time, we include a most important element of practical utility, which applies directly to the dearest interests of life. Ours is an association not only for pleasure, but for mutual instruction, protection, and profit.

Through the mutual cooperation of all we secure in a large measure the comfort and happines of each individual member.

An association of the character and for these purposes brings into play the noblest and best sentiments of our human nature. We find here an influence and an element which protects our profession, which fosters and encourages the younger members of our profession, which secures domestic happiness and lends its aid to all noble, social, and civil relations of life.

We are in the world to make the world better, to lift it up to the higher levels of enjoyment and progress, and to make hearts and homes brighter and happier by devoting to our fellow beings our best thoughts, activities, and influences.

